# Bioactive glass nanoparticles induce intrinsic *p53*-dependent apoptosis and promote genomic instability via ROS overproduction and mitochondrial depolarization in triple-negative breast cancer cells

**DOI:** 10.1038/s41598-025-32827-9

**Published:** 2026-01-06

**Authors:** Hanan R. H. Mohamed, Mayada E. Borai, Shahd Mosaad, Aya A. Osman, Alaa H. Elsewedy, Habiba M. Zaki, Ayman Diab, Gehan Safwat

**Affiliations:** 1 Department of Zoology, Faculty of Science, Cairo University, Giza, Egypt; 2 Faculty of Biotechnology, October University for Modern Sciences and Arts (MSA), 6th of October City, Egypt

**Keywords:** BGNps, TNBC, MDA-MB-231 cancer cells, Cytotoxicity, Oxidative stress, Genomic DNA integrity, Mitochondrial deplorization and apoptosis induction, Biochemistry, Biotechnology, Cancer, Cell biology, Drug discovery, Molecular biology

## Abstract

Triple-negative breast cancer (TNBC) is among the most aggressive breast cancer subtypes, characterized by the absence of estrogen receptor, progesterone receptor, and HER2 expression. The lack of these molecular targets, combined with the limitations of current treatment, particularly chemotherapy, which suffers from poor tumor selectivity, systemic toxicity, rapid development of resistance, and high recurrence rates, underscores the urgent need for innovative therapeutic strategies. Nanoparticle-based therapies have emerged as promising alternatives to overcome these challenges. Bioactive glass nanoparticles (BGNps), in particular, are recognized for their biocompatibility and multifunctional biological activity, yet their anticancer potential against TNBC remains fully unexplored. This study therefore aimed to investigate the therapeutic efficacy and molecular mechanisms of BGNps in highly aggressive triple-negative MDA-MB-231 breast cancer cells. Cells were treated with two-fold increasing concentrations of BGNps (7.8–1000 µg/ml), and cytotoxicity was assessed using the MTT assay. Genomic DNA integrity was evaluated using the alkaline comet assay, while oxidative stress and mitochondrial function were measured with 2′,7′-dichlorodihydrofluorescein diacetate (2′,7′-DCFH-DA) and Rhodamine-123 staining, respectively. Apoptotic induction was further examined using DAPI nuclear staining and chromatin diffusion assays, and transcriptional regulation of apoptosis- and mitochondria-related genes was analyzed by qRT-PCR. The results of MTT assay demonstrated that BGNps exerted potent, concentration-dependent cytotoxicity in MDA-MB-231 cells, with an IC50 value of 184.3 µg/ml. Treatment with BGNps at the IC50 concentration induced excessive reactive oxygen species (ROS) generation, severe mitochondrial membrane depolarization, extensive genomic DNA damage, and pronounced apoptotic cell death in MDA-MB-231 cancer cells. These effects were associated with marked upregulation of *p53* and concurrent downregulation of anti-apoptotic *Bcl-2* and mitochondrial *ND3* genes, amplifying oxidative stress and mitochondrial dysfunction. In conclusion, BGNps display strong potential as a novel nanotherapeutic for TNBC, offering an effective alternative to conventional chemotherapy. Their multi-step mechanism; encompassing ROS induction, mitochondrial disruption, and apoptosis activation, highlights their promise in overcoming the intrinsic resistance and therapeutic limitations of this highly aggressive breast cancer subtype.

## Introduction

Triple-negative breast cancer (TNBC) is an aggressive and clinically challenging subtype of breast cancer characterized by the absence of estrogen receptors, progesterone receptors, and human epidermal growth factor receptor 2 expression. This phenotype deprives TNBC patients of the therapeutic benefits offered by endocrine or HER2-targeted therapies, which are effective in other breast cancer subtypes. TNBC accounts for approximately 15–20% of all breast cancer cases worldwide and the global incidence of TNBC has been steadily rising, with disproportionately more prevalent among younger women, African-American populations, and individuals with BRCA1 mutations^1–3^. Despite its relatively lower incidence compared to other subtypes, TNBC is associated with a significantly higher risk of high proliferation rate, early metastasisspread, particularly to visceral organs and the brain, recurrence, and poor prognosis due to the lack of targeted therapies^4^.

Despite advances in oncology, systemic chemotherapy remains the primary treatment for TNBC due to the lack of specific molecular targets. Standard regimens typically include anthracyclines, taxanes, and platinum-based drugs. However, clinical outcomes remain unsatisfactory, especially in patients with advanced, recurrent, or metastatic disease, where survival rates are markedly lower and disease progression is often rapid^5–7^. Chemotherapy is further limited by poor tumor selectivity, systemic toxicity, and cumulative side effects such as cardiotoxicity, myelosuppression, and neurotoxicity, which compromise both treatment adherence and patient quality of life^6,8^. Furthermore, chemotherapy-induced genotoxic stress frequently leads to the development of chemoresistance, tumor recurrence, and long-term damage to healthy tissues^9–11^. These multifaceted challenges clearly highlight the urgent need for innovative therapeutic strategies that are safer, more selective, and mechanistically distinct from conventional chemotherapeutic agents that can selectively target TNBC and overcome chemoresistance while minimizing harm to healthy tissues.

Recent developments in nanotechnology offer promising strategies for enhancing cancer therapy through improved drug delivery, selective cytotoxicity, and controlled cellular interactions. Among these, bioactive glass nanoparticles (BGNps) have emerged as a novel class of inorganic nanomaterials with potential biomedical applications beyond bone regeneration due to their unique physicochemical properties, including high biocompatibility, controlled biodegradability, and ion-releasing capabilities^12–15^. BGNps are composed primarily of silicon, calcium, sodium and phosphorus oxides, and upon degradation in biological fluid, they release these therapeutic ions that can interact with cellular environments to modulate biological responses such as proliferation, mitochondrial function, apoptosis, and oxidative stress^16–19^. These properties suggest that BGNps may function not only as delivery vehicles, but also as standalone cytotoxic agents capable of disrupting tumor cell viability through non-drug-mediated mechanisms.

Although the regenerative utilities of BGNps in bone repair and tissue engineering are well documented^20,21^, their therapeutic potential in oncology, particularly in TNBC, remains fully unexplored. Emerging evidence suggests that bioactive glass particles exhibit selective cytotoxicity against certain malignancies, such as osteosarcoma and glioblastoma, primarily through the generation of reactive oxygen species (ROS), induction of genomic instability, and activation of apoptotic pathways^22,23^. However, these studies have utilized bulk or microparticulate forms of bioactive glass, which differ significantly from nanoparticles in terms of surface area-to-volume ratio, ion release kinetics, and cellular uptake behavior^15^. The biological interactions of BGNps with cancer cells, particularly their impact on mitochondrial membrane integrity, DNA stability, and ROS-mediated cytotoxic mechanisms in breast cancer cells, remain poorly characterized, especially within the context of TNBC, which is known for its aggressive phenotype and limited treatment options. Notably, TNBC and other cancer cells exhibit heightened susceptibility to oxidative stress and mitochondrial dysfunction compared to normal cells, suggesting a promising therapeutic window for BGNps. Despite this, systematic studies assessing BGNps’ impact on critical cellular processes such as cell viability, genomic stability, and mitochondrial permeability in TNBC models are lacking. This represents a significant knowledge gap and highlights the urgent need for mechanistic investigations into their potential as selective, non-drug-based anticancer agents.

To date, no published research has thoroughly examined the therapeutic effects of BGNps specifically in TNBC models. The absence of detailed mechanistic insights into their cytotoxicity, coupled with variability in nanoparticle synthesis, characterization, and assay methodologies, poses considerable obstacles to clinical translation^24–27^. Given the distinct physicochemical and biological behaviors of nanoparticles relative to their bulk counterparts, extrapolation from macroparticulate studies to nanoscale materials is unreliable without direct experimental validation. For addressing these critical gaps, the present study was conducted to explore the anticancer potential of BGNps against TNBC using the MDA-MB-231 cell line as a well-established in vitro model. Beyond evaluating general cytotoxicity, the current study aimed to investigate multiple mechanistic endpoints, including genomic DNA integrity, mitochondrial membrane potential disruption, intracellular ROS production, and apoptosis induction. Unlike prior studies focusing on BGNps as drug delivery platforms, this research uniquely examines their intrinsic cytotoxic properties, independent of chemotherapeutic loading. By elucidating the role of the ROS–mitochondria–DNA damage axis, the findings offer novel insights into BGNps’ anticancer mechanisms and support their future development as safe, selective, and innovative nanotherapeutics for triple-negative breast cancer.

## Materials and methods

### Chemicals

All chemicals used in this study were of high molecular-grade purity, particularly BGNps were procured from Nanotech Company (6th of October City, Cairo, Egypt) in fine white powdered form. According to the manufacturer’s specifications, the BGNps have a molar composition of 53% SiO₂ and 6% CaO/P₂O₅ (w/w), consistent with typical 53S6-type bioactive glass formulations. To prepare the tested concentrations, BGNps were suspended in dimethyl sulfoxide (DMSO; Sigma-Aldrich, USA) and ultrasonicated for 15–20 min to ensure homogeneous dispersion and prevent agglomeration. Essential reagents, including DMSO, 3-(4,5-dimethylthiazol-2-yl)-2,5-diphenyl tetrazolium bromide (MTT), and trypan blue dye, were sourced from Sigma-Aldrich (St. Louis, MO, USA).

Cell culture supplies, including Dulbecco’s Modified Eagle Medium (DMEM), HEPES buffer, L-glutamine, gentamycin, and Trypsin-EDTA, were purchased from Lonza (Belgium). Media were supplemented with 10% fetal bovine serum (FBS) and 1% gentamycin, while phenol red-free formulations were used to avoid spectrophotometric interference. All reagents were freshly prepared, and experimental work was conducted under aseptic conditions in a Class II biosafety cabinet to maintain sterility and reproducibility.

### Characterization of tested BGNps

The BGNps used in this study were characterized using complementary physicochemical techniques to assess structure, size, surface charge, and morphology. X-ray diffraction (XRD) analysis was performed using an XPERT-PRO diffractometer (PANalytical, Netherlands) equipped with Cu Kα radiation (λ = 1.5406 Å) to evaluate the crystalline versus amorphous nature of the particles. The obtained diffraction patterns were analyzed for characteristic peaks indicative of specific crystalline phases or broad halos representative of amorphous glassy structures. Particle size distribution and surface charge characteristics were determined using dynamic light scattering (DLS) on a Malvern Zetasizer Nano Series (Malvern Instruments, USA). DLS measurements provided the hydrodynamic diameter, reflecting particle size in suspension, while zeta potential values were used to assess colloidal stability and predict the likelihood of aggregation. These parameters are critical for understanding nanoparticle dispersion behavior and their interactions with biological systems.

Morphological features were examined using transmission electron microscopy (TEM) with a Tecnai G20 Super Twin microscope (FEI, USA) operating at an accelerating voltage of 200 kV. For sample preparation, a drop of BGNps suspension was carefully deposited onto carbon-coated copper grids, followed by air-drying at room temperature to preserve particle integrity. TEM imaging allowed direct visualization of particle shape, size, and aggregation state at the nanoscale, enabling cross-validation of the size data obtained from DLS. The combination of XRD, DLS, and TEM provided a comprehensive characterization of BGNps, ensuring a precise understanding of their physical attributes before their application in subsequent biological experiments.

### Propagation and cultivation of MDA-MB-231 cells

Human MDA-MB-231 breast cancer cells, a widely validated triple-negative breast cancer (TNBC) model, were purchased from the Regional Center for Mycology and Biotechnology (RCMB), Al-Azhar University, Cairo, Egypt, which provides authenticated and contamination-free cell lines. The cell line was supplied with a certificate of analysis confirming identity (STR profiling) and mycoplasma-free status. The use of this commercially available cell line does not require institutional ethical approval; however, all procedures were conducted in accordance with the institutional biosafety regulations of our laboratory.Cells were cultured in high-glucose DMEM (4.5 g/L) supplemented with 10% heat-inactivated fetal bovine serum (FBS), 50 µg/mL gentamycin, 100 U/mL penicillin, and 100 µg/mL streptomycin. Cultures were maintained at 37 °C in a humidified incubator with 5% CO_2_. The medium was replaced every 2–3 days, and cells were subcultured 2–3 times per week using 0.25% trypsin-EDTA once they reached 70–80% confluence. Only healthy, exponentially growing cells exhibiting ≥ 90% viability were used for all experimental assays.

### Estimation of BGNps cytotoxicity in MDA-MB-231 cells

The cytotoxic potential of BGNps on human MDA-MB-231 cells, a well-established TNBC model, was detected using the MTT assay according to the previously designed protocol^29,30^. MDA-MB-231 cells were cultured into sterile 96-well flat-bottom plates (Falcon, NJ, USA) at a density of 1 × 10^4^ cells per well in 100 µL of complete DMEM supplemented with 10% heat-inactivated FBS, 1% L-glutamine, 100 U/mL penicillin, and 100 µg/mL streptomycin. After 24 h of incubation at 37 °C in a humidified 5% CO₂ atmosphere to allow cell adhesion, the medium was replaced with fresh DMEM containing serial two-fold dilutions of BGNps (7.8, 15.6, 31.25, 62.50, 125, 250, 500 and 1000 µg/ml). Each concentration was tested in triplicate, with control wells receiving medium without BGNps.

Following 72 h of treatment with BGNps, the medium was removed and replaced with 100 µL of phenol red-free DMEM. Subsequently, 10 µL of 12 mM MTT stock solution (5 mg/mL in PBS) was added to each well. Plates were incubated for 4 h at 37 °C in the dark to allow viable cells to reduce MTT to insoluble purple formazan crystals. After incubation, 85 µL of the medium was gently removed, and 50 µL of DMSO was added to dissolve the formazan. Plates were incubated for an additional 10 min at 37 °C, and absorbance was measured at 590 nm using a microplate reader (SunRise, TECAN, USA). Cell viability (%) was calculated using the formula: Cell viability (%) = OD/ODc× 100.

where ODₜ is the mean absorbance of treated wells and OD is the mean absorbance of control wells. The IC50 values were determined by non-linear regression analysis using GraphPad Prism (San Diego, CA, USA) based on three independent experiments. Results were expressed as mean ± standard deviation (SD).

### Treatment schedule for MDA-MB-231 cells

Human MDA-MB-231 cells were cultured in T25 flasks containing DMEM medium supplemented with 1% L-glutamine, 10% heat-inactivated FBS, and antibiotics (100 µg/mL streptomycin and 100 U/mL penicillin). Cultures were maintained at 37 °C in a humidified incubator with 5% CO₂ until cells reached approximately 70–80% confluence. At this stage, cells were divided into untreated control and BGNps-treated cells: Untreated control cells were received culture medium containing < 0.1% DMSO, while BGNps-treated cells were exposed to BGNps at their predetermined IC50 concentration from the MTT cytotoxicity assay. Both untreated and BGNps-treated cells were incubated for 72 h under identical culture conditions. After treatment, cells were detached using 0.25% trypsin-EDTA, collected by centrifugation at 1,500 rpm for 5 min at 4 °C, and washed twice with ice-cold phosphate-buffered saline (PBS, pH 7.4) to remove any remaining culture medium or nanoparticles. The resulting cell pellets were resuspended in PBS and stored at − 80 °C for subsequent molecular and biochemical analyses. All experimental treatments were conducted in triplicate to ensure reproducibility and statistical accuracy.

### Detection of genomic DNA integrity in MDA-MB-231 cells

The genomic DNA integrity and possible induction of genomic DNA damage in human triple negative MDA-MB-231 breast cancer cells following treatment with BGNps at their predetermined IC50 concentration for 72 h was evaluated using the alkaline single-cell comet assay, according to the standardized protocols of Tice et al.^31^ and Langie et al.^32^. Briefly, approximately 10,000 cells in 15 µL of MDA-MB-231 cell suspension were mixed with 60 µL of 0.5% low-melting-point agarose (prepared in PBS at 37 °C) and immediately spread onto clean microscope slides pre-coated with 1% normal-melting-point agarose. Slides were allowed to solidify at room temperature for 30 min, then immersed in cold lysis buffer (2.5 M NaCl, 100 mM EDTA, 10 mM Tris-HCl, pH 10) supplemented with 1% Triton X-100 and 10% DMSO, and incubated for 24 h at 4 °C in the dark to minimize artifactual DNA damage. Following lysis, slides were transferred to freshly prepared alkaline electrophoresis buffer (300 mM NaOH, 1 mM EDTA, pH > 12) for 15 min to allow DNA unwinding. Electrophoresis was then performed at 25 V and 300 mA for 30 min at 4 °C. After electrophoresis, slides were neutralized in 0.4 M Tris-HCl buffer (pH 7.5) for 5 min, fixed in cold ethanol for 5 min, and air-dried. DNA was stained with 50 µL of ethidium bromide (20 µg/mL) and visualized under a fluorescence microscope. For each sample, fifty randomly selected nuclei were captured and analyzed using COMETSCORE™ software. DNA damage was quantified using three standard comet assay indices: tail length (distance of DNA migration), % DNA in tail (proportion of DNA present in the comet tail), and tail moment (product of tail length and % DNA in tail). Data are presented as mean ± SD from three independent experiments, with statistical analysis applied to determine the significance of BGNP-induced genotoxicity in MDA-MB-231 cells.

### Fluorometric determination of ROS generation in MDA-MB-231 cancer cells

The impact of BGNps exposure on intracellular ROS production in human triple-negative MDA-MB-231 breast cancer cells was assessed following 72 h of treatment with BGNps at their predetermined IC₅₀ concentration. Quantification of ROS was performed using the cell-permeable fluorescent probe 2′,7′-dichlorofluorescin diacetate (2,7-DCFH-DA), following the standardized protocol described by Siddiqui et al.^33^. Briefly, equal volumes of MDA-MB-231 cell suspension and 20 µM 2,7-DCFH-DA solution were gently mixed in sterile microcentrifuge tubes and incubated in the dark at room temperature for 30 min. Upon entering the cells, 2,7-DCFH-DA was enzymatically deacetylated by intracellular esterases to yield non-fluorescent 2,7-dichlorofluorescin (DCFH), which was subsequently oxidized by ROS to form the highly fluorescent compound 2,7-dichlorofluorescein (DCF). The green fluorescence intensity emitted by DCF was directly proportional to intracellular ROS levels. Following incubation, stained cells were carefully spread as a thin monolayer on clean, pre-labeled glass microscope slides and examined under an epifluorescence microscope equipped with filters optimized for DCF detection. Fluorescent images were captured at 200× magnification from randomly selected fields, ensuring identical exposure settings across all samples to allow for accurate comparison. The fluorescence intensity, representing intracellular ROS production, was quantified using Fiji (ImageJ) software, and relative ROS levels were determined by comparison between BGNps-treated and untreated control cells. All assays were conducted in triplicate to ensure reproducibility and statistical robustness of the data.

### Estimation of mitochondrial membrane integrity in MDA-MB-231 cancer cells

The influence of BGNps on mitochondrial membrane potential integrity, a vital marker of mitochondrial function and an early indicator of apoptosis, was quantitatively assessed in triple-negative MDA-MB-231 breast cancer cells after 72 h of exposure to BGNps at their predetermined IC50 concentration. The analysis utilized Rhodamine-123, a cationic, cell-permeable fluorescent dye that selectively accumulates within active, polarized mitochondria. The experimental procedure, adapted from Zhang et al.^34^, involved combining equal volumes of the cell suspension with a Rhodamine-123 working solution (10 µg/mL) in sterile, light-protected microcentrifuge tubes, followed by incubation at 37 °C for 1 h in the dark to facilitate dye uptake. After incubation, cells were washed twice with ice-cold PBS to remove residual dye and reduce background fluorescence.

The stained cells were then mounted onto pre-cleaned glass slides, arranged into a uniform monolayer, and covered with sterile coverslips. Cell Imaging was performed using an epifluorescence microscope equipped with Rhodamine-123-specific filters, capturing images at 200× magnification from randomly selected fields under identical exposure settings for all samples. Fluorescence intensity, reflecting mitochondrial polarization, was quantified using Fiji (ImageJ) software. A pronounced decrease in Rhodamine-123 fluorescence in BGNps-treated cells relative to untreated controls indicated substantial mitochondrial depolarization, signifying early mitochondrial impairment and the onset of apoptosis. All measurements were conducted in triplicate, and results were expressed as mean ± SD to ensure reproducibility and statistical reliability.

### Screening apoptosis induction in MDA-MB-231 cancer cells

Apoptosis, the programmed elimination of damaged or malignant cells, is a central mechanism in anticancer therapy. Its detection provides vital insight into the cytotoxic action of experimental agents. In this study, the induction of apoptosis in triple-negative MDA-MB-231 breast cancer cells was assessed following a 72-hour exposure to BGNps at their IC50 concentration. Untreated cells served as negative controls. Two complementary approaches were employed to ensure robust detection: (i) the chromatin diffusion assay, enabling the identification of DNA fragmentation, a hallmark of late-stage apoptosis, and (ii) nuclear staining with 4′,6-diamidino-2-phenylindole (DAPI) to visualize characteristic apoptotic nuclear alterations, including chromatin condensation and nuclear fragmentation.

#### Chromatin diffusion assay

The chromatin diffusion assay relies on the presence of alkali-labile sites in apoptotic DNA, which fragment and diffuse under alkaline conditions within an agarose matrix, producing a distinct halo around the nucleus^35^. Slides were first pre-coated with 0.7% normal-melting-point agarose. MDA-MB-231 cells were suspended in low-melting-point agarose and layered onto the coated slides. After air drying, slides underwent cold lysis to remove cytoplasmic contents, leaving intact nuclear DNA. Following neutralization with Tris buffer and fixation in cold ethanol, slides were stained with ethidium bromide. Under a fluorescence microscope, cells with DNA halos were scored as apoptotic. For each sample, 1000 cells were examined, and apoptotic percentages were calculated to quantitatively assess the extent of DNA fragmentation.

#### DAPI nuclear staining

DAPI, a DNA-binding fluorescent dye, was used to confirm apoptosis by detecting chromatin condensation and nuclear fragmentation^36^. Triple-negative MDA-MB-231 breast cancer cells were seeded into 96-well plates at a density of 1 × 10⁴ cells per well and allowed to adhere overnight. Cells were then exposed to BGNps at their IC50 concentration for 72 h. Following treatment, cells were gently washed with PBS to remove residual medium, fixed with 4% paraformaldehyde for 15 min at room temperature, and stained with DAPI (1 µg/mL in PBS) for 1 h in the dark to ensure optimal nuclear labeling. Fluorescence imaging was performed using an epifluorescence microscope equipped with a DAPI filter set at 200× magnification. Apoptotic nuclei were identified by their bright, condensed fluorescence and fragmented appearance, whereas healthy nuclei exhibited uniform, diffuse staining. For quantitative assessment, a total of 1,000 cells per group were examined, and the proportion of apoptotic cells was calculated.

### Quantification of p53, ND3 and Bcl2 gene expression in MDA-MB-231 cancer cells

To further elucidate BGNps’ molecular impact on mitochondrial function and apoptosis in MDA-MB-231 cells, quantitative real-time PCR (qRT-PCR) was employed to measure mRNA expression of the pro-apoptotic *p53* gene, the mitochondrial *ND3* gene (a component of the electron transport chain), and the anti-apoptotic *Bcl-2* gene. Untreated control and BGNps-treated MDA-MB-231 cells were processed for total RNA extraction using the GeneJET RNA Purification Kit (Thermo Fisher Scientific, USA). RNA concentration and purity were confirmed with a NanoDrop spectrophotometer. One microgram of RNA from each sample was reverse-transcribed into complementary DNA (cDNA) using the High-Capacity cDNA Reverse Transcription Kit (Applied Biosystems, USA). qRT-PCR reactions were performed with SYBR Green PCR Master Mix and gene-specific primers listed in Table 1^37–39^ on a StepOnePlus Real-Time PCR System. GAPDH served as the internal control. Relative expression of the tested genes (*p53*,* ND3* and *Bcl2)* was determined using the comparative Ct (2⁻ΔΔCt) method. All reactions were run in triplicate, and results were expressed as mean ± SD.

**Table 1:** Sequences of primers used in qRT-PCR.

Gene	Strand	Primer’s sequences
GAPDH	Forward	5’-GAAGGTGAAGGTCGGAGTCA-3’
	Reverse	5’-GAAGATGGTGATGGGATTTC-3’
ND3	Forward	5’-CGCCGCCTGATACTGGCAT-3’
	Reverse	5’-CTAGTATTCCTAGAAGTGAG-3’
BCL-2	Forward	5’-TCCGATCAGGAAGGCTAGAGT-3’
	Reverse	5’-TCGGTCTCCTAAAAGCAGGC-3’
P53	Forward	5’-CAGCCAAGTCTGTGACTTGCACGTAC-3’
	Reverse	5’-CTATGTCGAAAAGTGTTTCTGTCATC-3’

### Statistical analysis

Data from this study were analyzed using the Statistical Package for the Social Sciences (SPSS). Results from the alkaline Comet assay, qRT-PCR, mitochondrial membrane integrity assessment, ROS generation, and apoptosis detection were expressed as mean ± SD. Statistical comparisons between BGNps-treated and untreated control MDA-MB-231 triple-negative breast cancer cells were performed using an unpaired two-tailed Student’s t-test, with statistical significance set at *p* < 0.05.

## Results

### Characterization of the used BGNps

**Fig. 1:**
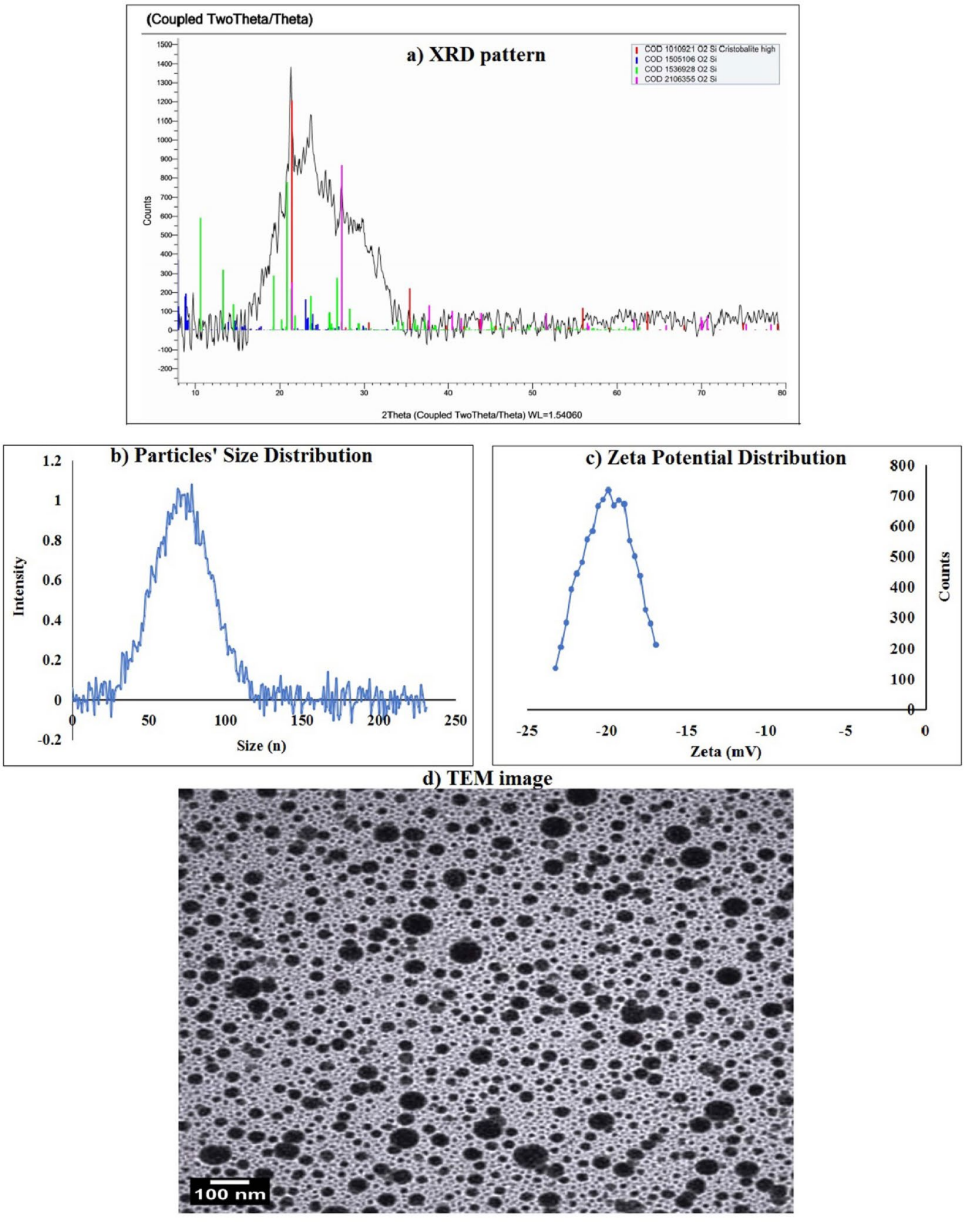
Characterization of BGNps showing (**a**) XRD profile, (**b**) Particles’ Size Distribution, (**c**) Zeta Potential Distribution and (**d**) Particles’ imaging using TEM microscopy.

Characterization using XRD analysis showed that the tested BGNps possessed an amorphous glass-like structure. This was indicated by a broad diffuse halo in the diffraction pattern within the theta angle range of 15.48°–35.11°, with peaks detected at 15.48°, 17.22°, 21.63°, 24.06°, 27.84°, 30.55°, and 35.11° (Fig. 1). The absence of sharp crystalline peaks confirms the lack of long-range order, a typical feature of bioactive glasses, which is often linked to increased surface reactivity and biological performance. Indeed, DLS analysis revealed that the particle sizes of the used BGNps ranged from 35.33 to 105.99 nm, with an average hydrodynamic diameter of 73.3 nm (Fig. 1), and the polydispersity index (PDI) value of 0.27, indicating a uniform and narrow size distribution of BGNps suspension. Imaging using TEM further confirmed that the BGNps were mostly spherical, evenly dispersed, and had smooth surfaces with minimal aggregation as seen in Fig. 1. The average particle diameter measured by TEM was about 31.5 nm, consistent with the nanoscale size determined by DLS.

### BGNps cause marked concentration-dependent death of MDA-MB-231 cancer cells

**Fig. 2:**
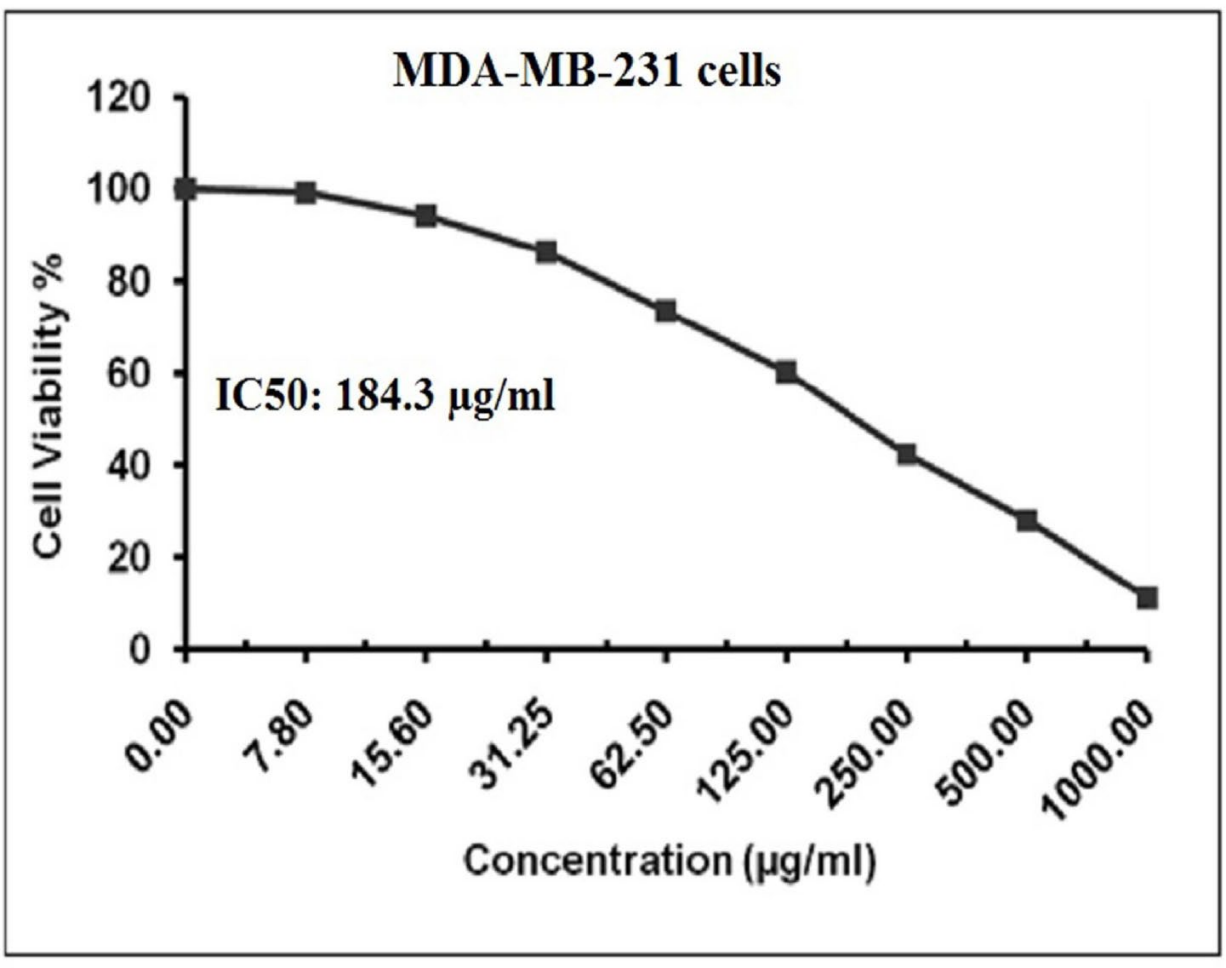
Viability of human triple negative MDA-MB-231 breast cancer cells assessed using the MTT assay after 72-hour exposure to BGNps at two-fold increasing concentrations (7.8, 15.6, 31.25, 62.5, 125, 250, 500 and 1000 µg/ml).

Interpretation of MTT assay results demonstrated a pronounced, concentration-dependent decreases in the viability of triple negative MDA-MB-231 cancer cells following 72 h exposure to multiple increasing concentrations of BGNps (7.8, 15.6, 31.25, 62.5, 125, 250, 500, and 1000 µg/ml), as depicted in Fig. 2; The calculated IC50 value was found to be 184.3 µg/ml, confirming the strong cytotoxic potential of BGNps against this aggressive cancer cells. These results consequently highlight the therapeutic potential of BGNps against the triple-negative MDA-MB-231 cancer cells and necessitate further mechanistic studies at the IC50 concentration to explore their underlying mode of BGNps action.

### BGNps cause dramatic genomic instability in MDA-MB-231 cancer cells

**Fig. 3:**
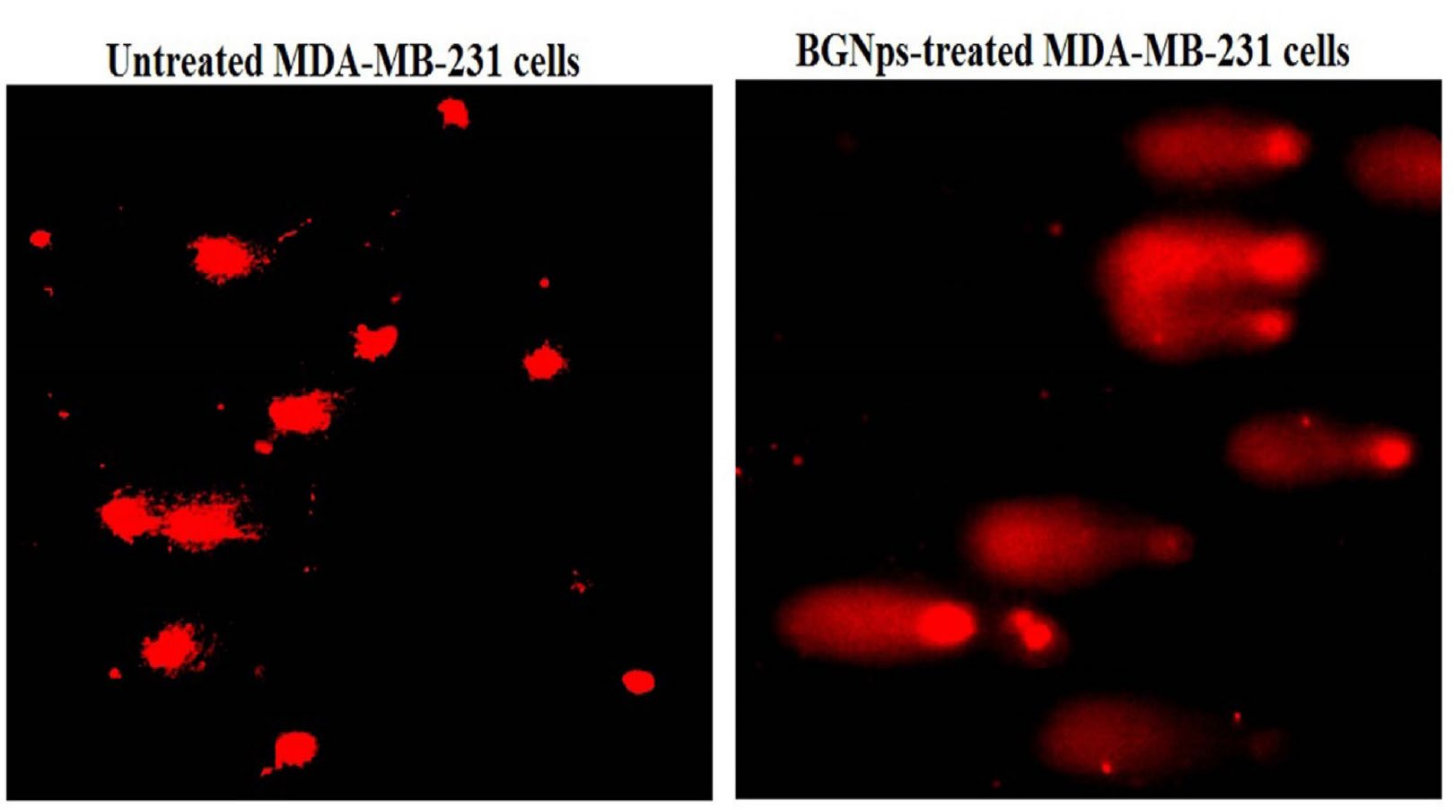
Examples for the scored Comet nuclei with intact DNA in untreated MDA-MB-231 breast cancer cells and those with damaged DNA in MDA-MB-231 breast cancer cells treated with the IC50 concentration of BGNps (184.3 µg/ml) for 72 h. Magnification 200x.

Screening the results of the alkaline Comet assay revealed a dramatic loss of genomic stability and high DNA damage in highly aggressive triple negative MDA-MB-231 breast cancer cells after 72 h exposure to BGNps at the IC50 concentration of 184.3 µg/ml. This marked genotoxic effect was manifested by the statistically significant increases (*p* < 0.001) in main Comet assay parameters: tail length, %DNA in the tail, and tail moment compared to their measured values in the untreated control MDA-MB-231 cancer cells, as displayed in Table 2 and seen in Fig. 3. These parameters serve as well-established indicators of DNA strand breaks and fragmentation, confirming the potent genotoxic impact of BGNps in MDA-MB-231 cancer cells under the experimental conditions. Microscopic examination shown in Fig. 3 further supported these results through the appearance of control MDA-MB-231 cells with compact dense nuclei and minimal DNA migration, and the BGNps-treated cells exhibited pronounced comet tails, hallmarks of severe DNA damage. Collectively, the quantitative data and visual comet patterns clearly demonstrate the strong DNA-damaging potential of BGNps in MDA-MB-231 cancer cells.

**Table 2:** Genomic DNA damage in human triple-negative MDA-MB-231 breast cancer cells assessed by alkaline comet assay after 72 h exposure to IC50 concentration (184.3 µg/ml) of BGNps.

	Treatment (Concentration)	Tail length (px)	%DNA in tail	Tail moment
MDA-MB-231 cancer cells	Untreated (0.00 µg/ml)	4.21 ± 0.54	21.03 ± 1.10	1.15 ± 0.04
	BGNPs-treated (187.81 µg/ml)	18.39 ± 1.64 ^***^	37.79 ± 1.60 ^***^	6.91 ± 0.36 ^***^

• Results are expressed as mean ± SD.

• ***: Indicates statistical significant difference from the compared untreated control cells at *p* < 0.001, using independent student t-test.

### BGNps induce excessive ROS generation in MDA-MB-231 breast cancer cells

**Fig. 4:**
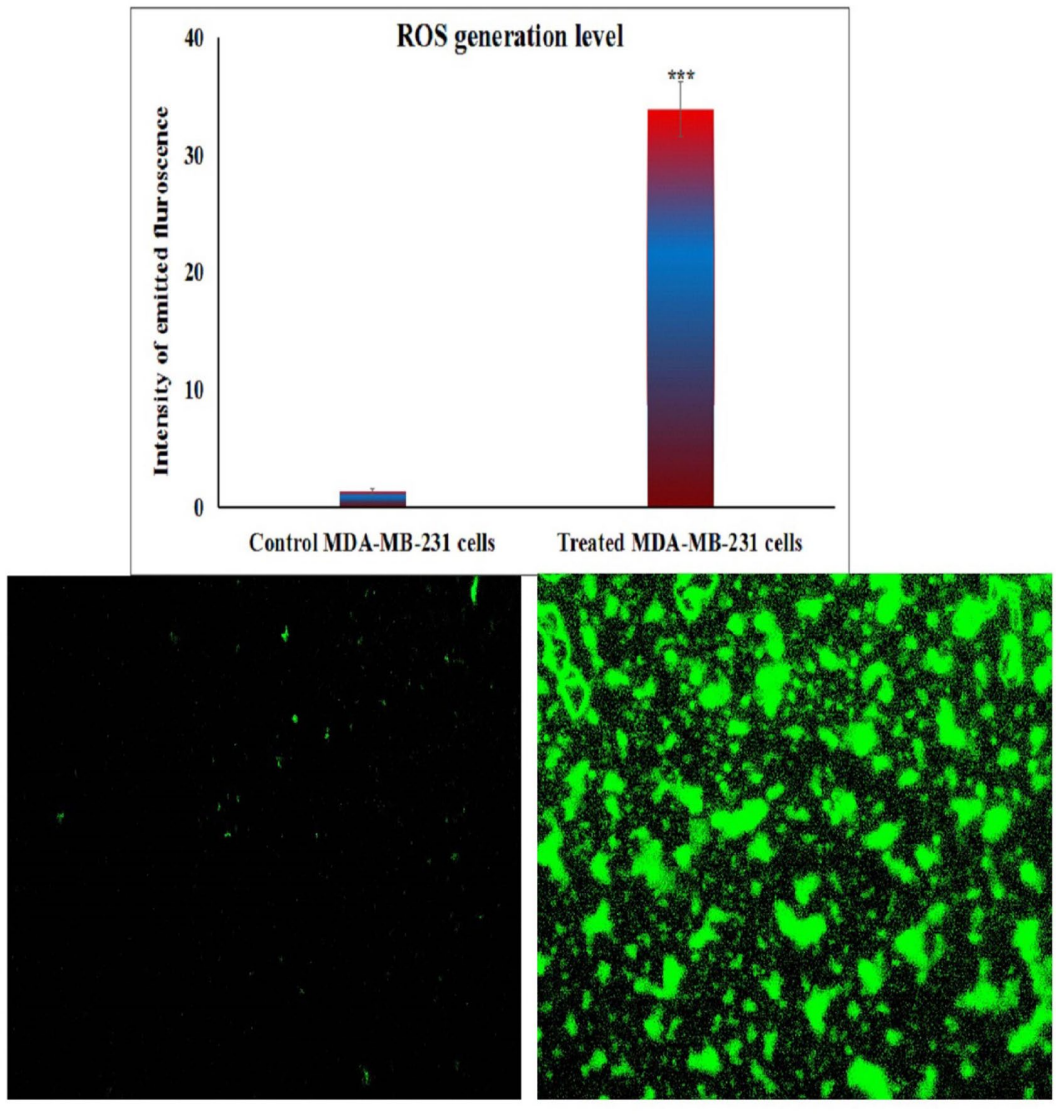
Generation level of ROS within the untreated and treated triple negative MDA-MB-231 breast cancer cells with the IC50 concentration (184.3 µg/ml) of BGNps for 72 h. Magnification 200x.

As shown in Fig. 4, exposure of triple-negative MDA-MB-231 breast cancer cells to BGNps at the IC50 concentration (184.3 µg/ml) for 72 h resulted in a significant elevation of intracellular ROS level. This oxidative burst was quantitatively confirmed by a highly significant increase (*p* < 0.001) in the fluorescence intensity of the ROS-sensitive probe 2′,7′-DCFH-DA, which fluoresces upon oxidation by ROS. Compared with untreated control cells, BGNps-treated cells displayed markedly stronger fluorescence signals, reflecting extensive cytoplasmic ROS accumulation. These findings clearly demonstrate that BGNps trigger oxidative stress in triple-negative MDA-MB-231 cancer cells.

### BGNps severely disrupt mitochondrial membrane potential in MDA-MB-231 breast cancer cells

**Fig. 5:**
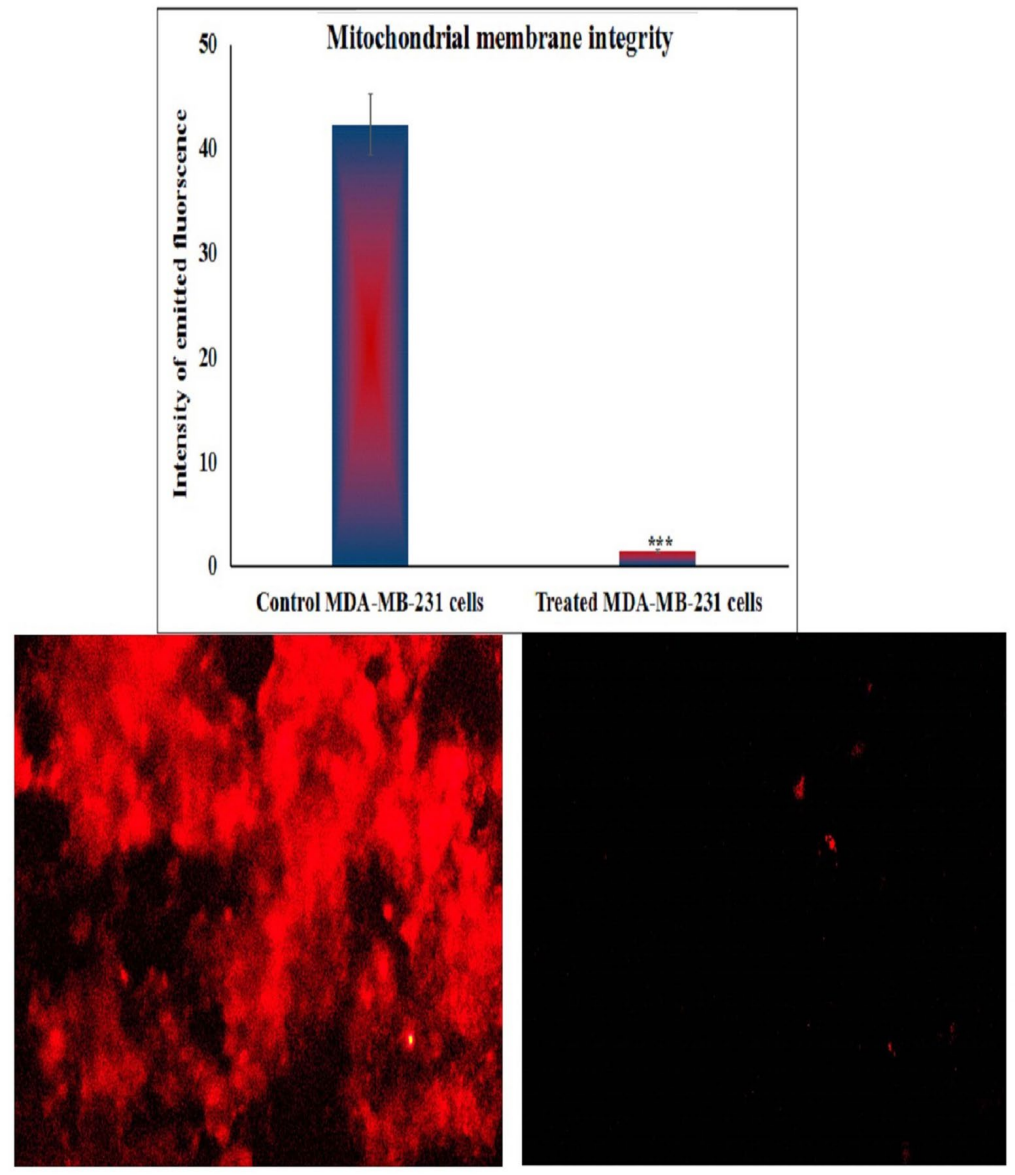
Mitochondrial membrane potential integrity in the untreated and treated triple negative MDA-MB-231 breast cancer cells with the IC50 concentration (184.3 µg/ml) of BGNps for 72 h. Magnification 200x.

Staining of triple negative MDA-MB-231 breast cancer cells with Rhodamine-123, a highly selective cationic dye for active mitochondria with intact membrane potential, demonstrated a dramatic loss of mitochondrial membrane potential integrity in MDA-MB-231 breast cancer cells following 72 h of exposure to BGNps at the IC50 concentration (184.3 µg/ml). As presented in Fig. 5, this marked disruption was evidenced by a highly significant reduction (*p* < 0.001) in Rhodamine-123 fluorescence intensity in BGNps-treated cells compared with untreated control cells. The diminished fluorescence reflects mitochondrial depolarization and indicates substantial impairment of mitochondrial function and structural integrity in BGNps-treated MDA-MB-231 cells.

### BGNps induce pronounced apoptotic death in MDA-MB-231 breast cancer cells

**Fig. 6:**
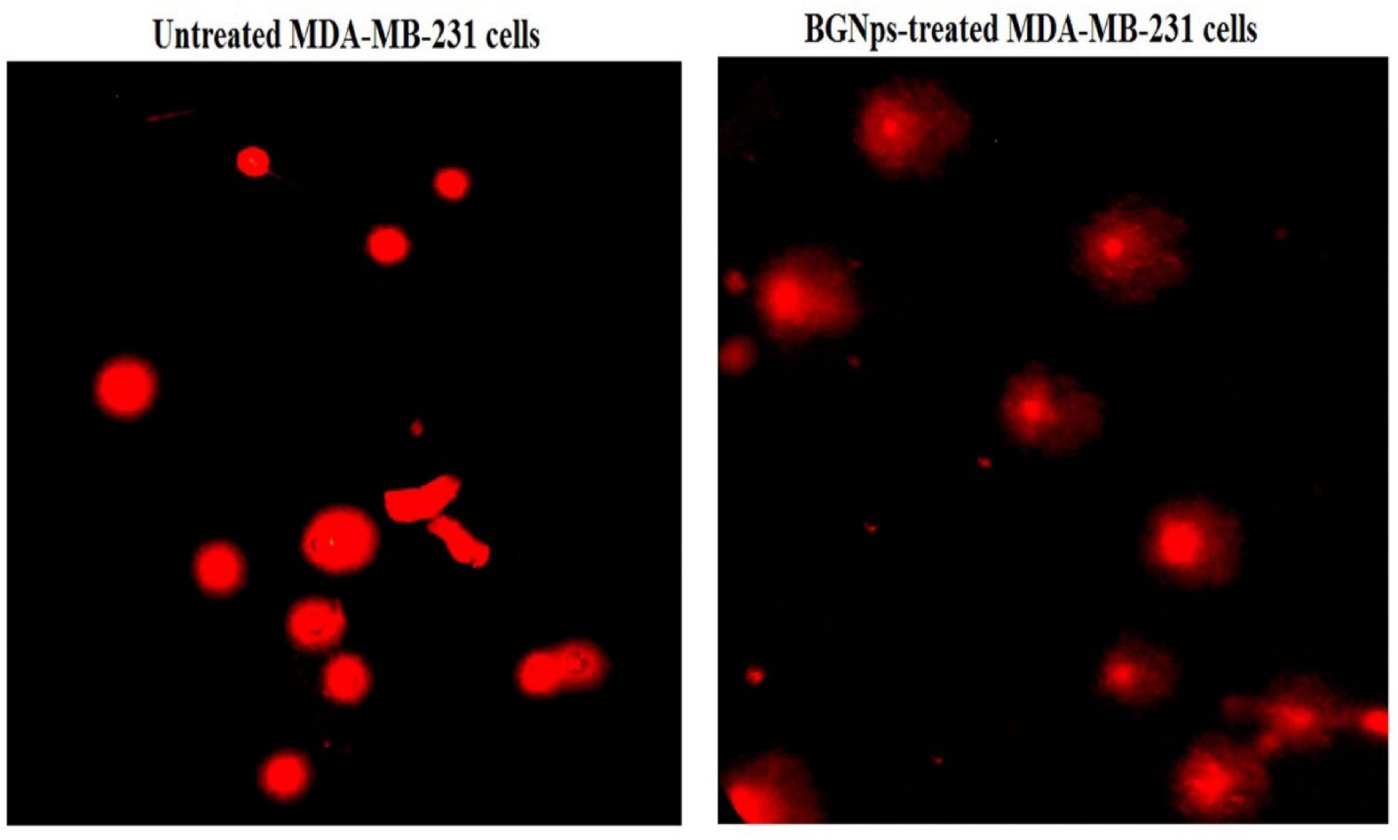
Difference between normal cells with intact DNA and apoptotic cells exhibiting diffused DNA using Chromatin diffusion assay in untreated and BGNps-treated triple negative MDA-MB-231 breast cancer cells following 72-hour exposure to the IC50 concentration (184.3 µg/ml). Magnification: 200×.

Chromatin diffusion assay and DAPI nuclear staining revealed a significant induction of apoptosis in triple-negative MDA-MB-231 breast cancer cells after 72 h of exposure to the IC50 concentration (184.3 µg/ml) of BGNps. This pronounced apoptotic response was characterized by abnormal nuclear morphology and extensive chromatin fragmentation as depicted in Figs. 5 and 6. In the chromatin diffusion assay, apoptotic cells were identified by distinct chromatin halos, indicating the cytoplasmic dispersion of fragmented DNA. Consistently, BGNps-treated cells exhibited prominent halos surrounding condensed nuclei, in contrast to the compact, intact nuclei of untreated control cells as seen in Fig. 6. Quantitative analysis confirmed highly significant increases (*p* < 0.001) in both the number of chromatin halos and the percentage of apoptotic cells as displayed in Table 3.

**Table 3:** Frequency of apoptosis induction in human triple-negative MDA-MB-231 breast cancer cells assessed by chromatin diffusion assay after 72 h exposure to IC50 concentration (184.3 µg/ml) of BGNps.

MDA-MB-231 cancer cells	Treatment (Concentration)	Number of cells with		Percentage of apoptotic cells
		Intact DNA	Diffused DNA	
	Untreated (0.00 µg/ml)	945.00 ± 6.08	555.00 ± 6.08	5.55 ± 0.61
	BGNPs-treated (187.81 µg/ml)	576.33 ± 30.92 ^***^	423.67 ± 30.92 ^***^	42.37 ± 3.09 ^***^

• Results are expressed as mean ± SD.

• ***: Indicates statistical significant difference from the compared untreated control cells at *p* < 0.001, using independent student t-test.

**Fig. 7:**
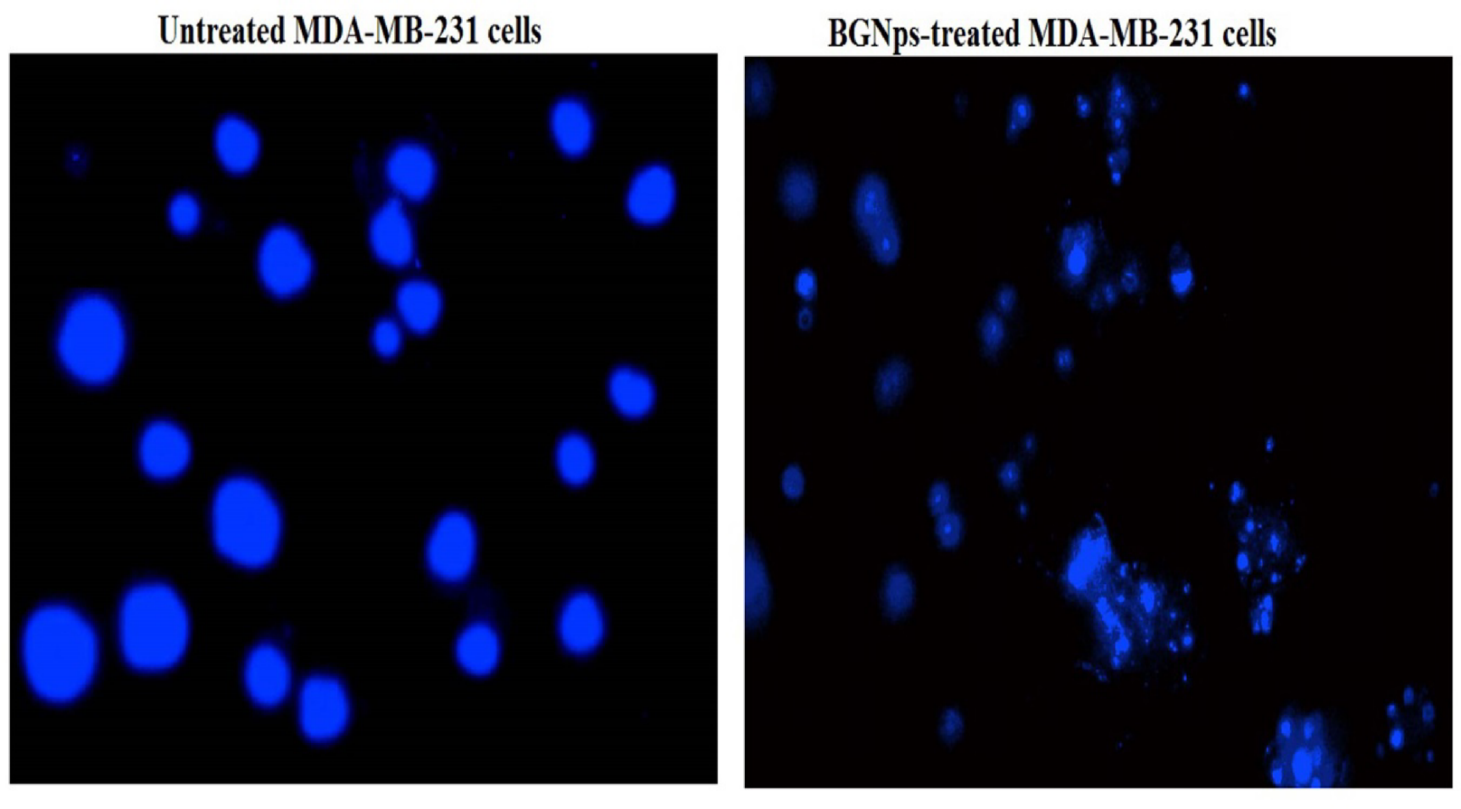
DAPI staining illustrating intact, uniformly stained nuclei in untreated MDA-MB-231 breast cancer cells and condensed or fragmented nuclei in apoptotic MDA-MB-231 breast cancer cells treated with the IC50 concentration (184.3 µg/ml) of BGNps for 72 h. Magnification: 200×.

DAPI staining further supported these findings by visualizing hallmark apoptotic nuclear changes. Untreated control MDA-MB-231 breast cancer cells displayed smooth, round, uniformly stained nuclei, whereas BGNps-treated cells exhibited chromatin condensation, nuclear shrinkage, fragmentation, and apoptotic bodies, evident as bright, condensed structures under fluorescence microscopy as shown in Fig. 7. Likewise, quantitative assessment showed a statistically significant rise (*p* < 0.001) in apoptotic nuclei following BGNps treatment compared with controls as noticed in Table 4. Collectively, these data confirm that BGNps trigger pronounced apoptosis in MDA-MB-231 breast cancer cells through chromatin dispersion and nuclear disintegration, highlighting their strong pro-apoptotic potential against this aggressive breast cancer subtype.

**Table 4:** Frequency of apoptosis induction in human triple-negative MDA-MB-231 breast cancer cells assessed by DAPI staining after 72 h exposure to IC50 concentration (184.3 µg/ml) of BGNps.

MDA-MB-231 cancer cells	Treatment (Concentration)	Number of cells with		Percentage of apoptotic cells
		intact DNA	fragmented and condensed DNA	
	Untreated (0.00 µg/ml)	922.67 ± 5.51	77.33 ± 5.51	7.73 ± 0.55
	BGNPs-treated (187.81 µg/ml)	503.33 ± 40.43 ^***^	496.67 ± 40.43 ^***^	49.67 ± 0.40 ^***^

• Results are expressed as mean ± SD.

• ***: Indicates statistical significant difference from the compared untreated control cells at p < 0.001, using independent student t-test.

### BGNps severely dysregulate the ***p53***,***ND3*** and ***Bcl2*** gene expression in MDA-MB-231 breast cancer cells

Results of qRT-PCR analysis demonstrated significant alterations in the expression of key apoptotic and mitochondrial regulatory genes in MDA-MB-231 breast cancer cells after 72 h of exposure to BGNps at the IC50 concentration (184.3 µg/ml) as displayed in Table 5. The pro-apoptotic gene *p53* was markedly upregulated (*p* < 0.001) in BGNps-treated cells compared with untreated control MDA-MB-231 breast cancer, indicating activation of *p53*-dependent apoptotic pathways. In contrast, both the mitochondrial gene *ND3* and the anti-apoptotic gene *Bcl2* were significantly downregulated (*p* < 0.001), reflecting impaired mitochondrial function and suppression of survival signaling. These transcriptional changes suggest that BGNps trigger mitochondrial dysfunction, oxidative stress, and a shift in the apoptotic balance toward cell death. The upregulation of *p53*, coupled with downregulation of *ND3* and *Bcl2*, highlights the ability of BGNps to disrupt mitochondrial homeostasis and promote intrinsic (mitochondrial-mediated) apoptosis in triple-negative MDA-MB-231 breast cancer cells.

**Table 5:** Fold change in the expression level of *p53*,* ND3* and *Bcl2* genes in human triple-negative MDA-MB-231 breast cancer cells after 72 h exposure to IC50 concentration (184.3 µg/ml) of BGNps.

	Treatment (Concentration)	p53	ND3	Bcl2
MDA-MB-231 cancer cells	Untreated (0.00 µg/ml)	1.00 ± 0.00	1.00 ± 0.00	1.00 ± 0.00
	BGNPs-treated (187.81 µg/ml)	4.33 ± 0.07 ^***^	0.77 ± 0.06 ^***^	0.04 ± 0.01 ^***^

• Results are expressed as mean ± SD.

• ***: Indicates statistical significant difference from the compared untreated control cells at *p* < 0.001, using independent student t-test.

## Discussion

Chemotherapy remains the standard treatment for triple-negative breast cancer (TNBC); however, its clinical benefits are often counterbalanced by severe systemic toxicities, rapid development of resistance, and high recurrence rates^40,41^. Patients commonly suffer from adverse toxic side effects such as myelosuppression, cardiotoxicity, neuropathy, and gastrointestinal complications, which not only impair quality of life but also limit treatment compliance^11,42,43^. Moreover, TNBC’s pronounced heterogeneity and the absence of defined molecular targets contribute to poor therapeutic outcomes and frequent relapse^7,44^. These limitations highlight the urgent need for novel, more targeted therapies that can selectively eradicate TNBC cells while minimizing harm to normal tissues.

Nanotechnology-based therapeutics offer promising alternatives, providing enhanced tumor selectivity, controlled delivery, and the potential to overcome resistance pathways^45,46^. In this regard, BGNps have gained increasing attention due to their excellent biocompatibility, tunable surface properties, and controlled release of therapeutic ions such as calcium, phosphate, and silicon, which are known to modulate essential cellular pathways^19,47^. While extensively applied in bone regeneration and antimicrobial therapy, their anticancer properties, particularly in TNBC, remain poorly defined. Accordingly, the present study explored the cytotoxic potential of BGNps against triple negative MDA-MB-231 breast cancer cells, with focus on estimating their ability to induce genomic instability, disrupt mitochondrial function, elevate ROS level, and trigger apoptosis in MDA-MB-231 cancer cells.

**Fig. 8:**
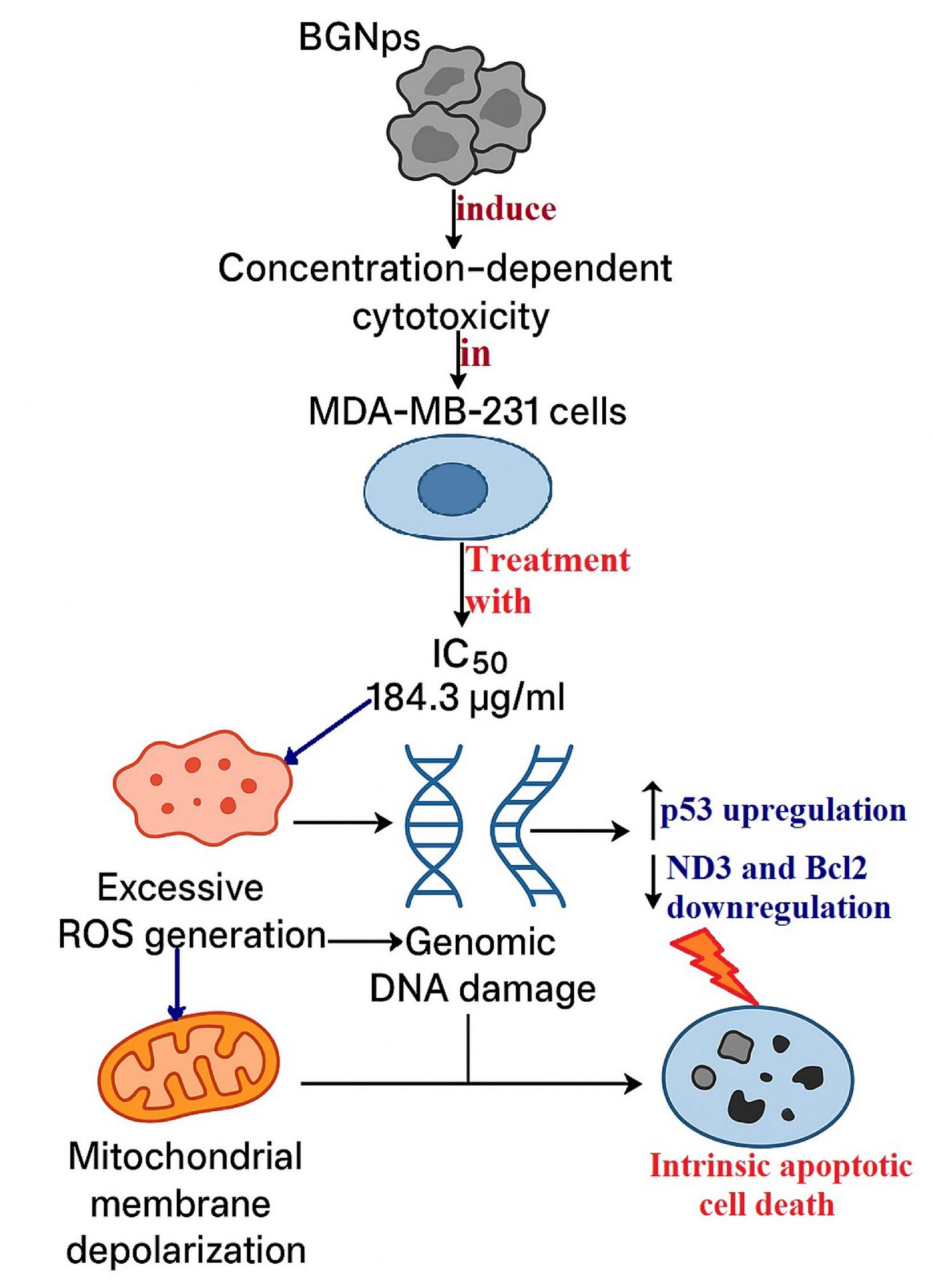
Mechanistic pathway of BGNps-induced intrinsic apoptosis in triple negative MDA-MB-231 breast cancer cells.

In this study BGNps exhibited potent, concentration-dependent cytotoxicity against triple negative MDA-MB-231 breast cancer cells, with an IC50 value of 184.3 µg/ml after 72 h of exposure to two-fold increasing concentration of BGNps ranging from 7.8 to 1000 µg/ml as displayed in Fig. 8. This pronounced cytotoxicity mirrors previous studies demonstrating the strong anticancer activity of bioactive glass-based formulations across various cancer models other than TNBC, where they were shown to effectively impair malignant cell viability while exerting comparatively lower toxicity toward non-cancerous cells. Such preferential activity has been largely attributed to the distinctive physicochemical features of BGNps, particularly their ability to release therapeutic ions and trigger cellular stress responses that disproportionately compromise rapidly dividing cancer cells^22,23^.

Mechanistically, BGNps triggered significant genomic DNA damage, as confirmed by the statistically significant increases in key alkaline Comet assay parameters; tail length, %DNA in tail and tail moment in highly aggressive MDA-MB-231 breast cancer cells, following 72 h of exposure to BGNps at the IC50 concentration value of 184.3 µg/ml. These alterations reflect extensive fragmentation of genomic DNA and are consistent with nanoparticle-induced oxidative stress and genotoxicity reported across several cancer models^48–50^. Given that DNA fragmentation is a defining feature of apoptosis, these results strongly suggest that BGNps initiate intrinsic apoptotic pathways in highly aggressive triple-negative MDA-MB-231 cells.

In addition to inducing genotoxic stress, our findings demonstrated pronounced mitochondrial dysfunction, a central event of the intrinsic apoptotic pathway. Screening mitochondrial membrane potential using Rhodamine-123, a mitochondrial membrane potential-sensitive dye, revealed a significant depolarization of mitochondrial membranes in BGNps-treated MDA-MB-231 breast cancer cells. This loss of mitochondrial integrity is mechanistically consistent with the role of ROS in damaging mitochondrial membranes, thereby promoting cytochrome c release and subsequent caspase activation^51,52^.

Furthermore, ROS measurement using 2′,7′-DCFH-DA confirmed a marked elevation in intracellular ROS generation level in BGNps-treated triple negative MDA-MB-231 breast cancer cells, reinforcing oxidative stress as a central mediator of BGNps-induced cytotoxicity. Notably, TNBC cells such as MDA-MB-231 are particularly vulnerable to oxidative injury due to their accelerated metabolic activity, elevated basal ROS production, and impaired oxidant defense capacity^53,54^. These intrinsic susceptibilities likely intensify BGNps-induced oxidative and mitochondrial damage, thereby amplifying their cytotoxic efficacy against this highly aggressive breast cancer subtype.

Collectively, as displayed in Fig. 8 the findings from this study demonstrate that BGNps exert potent pro-apoptotic effects in triple negative MDA-MB-231 breast cancer cells by inducing severe genomic DNA damage, oxidative stress, and mitochondrial dysfunction, ultimately driving the cells toward apoptotic cell death. Following 72-hour exposure to the IC50 concentration (184.3 µg/ml) of BGNps, chromatin diffusion assay and DAPI nuclear staining revealed a significant increase in apoptotic nuclei, confirming that apoptosis is the primary mode of cell death triggered by BGNps. Microscopic examination further validated this apoptotic response. BGNps-treated MDA-MB-231 cancer cells exhibited hallmark nuclear alterations, including chromatin condensation, nuclear shrinkage, and fragmentation, along with the appearance of apoptotic bodies, classic morphological signatures of programmed cell death^55,56^. On contrary, these changes were absent in untreated control cells, which retained round, intact nuclei with uniform chromatin distribution. Such nuclear abnormalities strongly support the activation of the intrinsic mitochondrial apoptotic pathway, a pathway often engaged when intracellular stress surpasses the protective capacity of survival mechanisms^57–59^.

The integration of our findings indicates that BGNps induce apoptosis in triple-negative MDA-MB-231 breast cancer cells through a coordinated, multi-step molecular mechanism. First, excessive ROS production overwhelms the inherently limited antioxidant defense capacity of TNBC cells, leading to extensive DNA strand breaks and severe genomic instability^60,61^. Second, ROS-mediated damage compromise mitochondrial membrane integrity, resulting in loss of mitochondrial membrane potential, cytochrome c release, and subsequent activation of caspase-dependent apoptotic cascades^55,62^. Third, this stress is further intensified by transcriptional dysregulation of apoptosis- and mitochondria-associated genes, which amplifies mitochondrial dysfunction and reinforces apoptotic signaling pathways^63–67^.

Consistently with these mechanisms, qRT-PCR analysis confirmed significant transcriptional reprogramming of apoptosis- and mitochondria-related genes in BGNps-treated MDA-MB-231 breast cancer cells. Specifically, p53 expression was markedly upregulated following 72 h of treatment at the IC50 concentration. Upregulation of p53 following BGNps treatment represents a crucial event in initiating programmed cell death. p53, the guardian of the genome, acts as a key tumor suppressor that responds to DNA damage and oxidative stress by activating downstream apoptotic pathways^63,68,69^. In TNBC, however, p53 is frequently mutated or functionally impaired, a defect strongly associated with therapeutic resistance^7,70^. Therefore, the observed reactivation or stabilization of p53 in MDA-MB-231 cells after BGNps exposure suggests that these nanoparticles may overcome defective p53 signaling and restore apoptotic competence. Once activated, p53 can induce apoptosis through transcription-dependent pathways, by upregulating pro-apoptotic genes, as well as transcription-independent mechanisms, including direct interactions with mitochondria and ROS-mediated signaling that promote cytochrome c release, mitochondrial depolarization, and dysfunction^71,72^.

Conversely, downregulation of Bcl2, a key anti-apoptotic gene, shifts the balance toward apoptosis. Bcl2 normally stabilizes mitochondrial membranes and prevents cytochrome c release by antagonizing pro-apoptotic members of the Bcl2 family^73^. Reduced expression of Bcl2 gene 72-hours following BGNps treatment compromises this protective barrier, thereby sensitizing mitochondria to depolarization and facilitating activation of the intrinsic apoptotic pathway. Notably, several studies have reported that nanoparticle-induced oxidative stress downregulates Bcl2 while upregulating pro-apoptotic proteins, reinforcing mitochondrial dysfunction and apoptosis^65–67,74,75^. In parallel, downregulation of ND3, a subunit of mitochondrial Complex I of the electron transport chain (ETC), further exacerbates mitochondrial stress. ND3 is essential for efficient electron transfer and ATP production. Its reduced expression disrupts ETC function, leading to electron leakage and accumulation of ROS^60^. Elevated ROS not only cause additional DNA damage but also amplify mitochondrial depolarization, establishing a feed-forward loop that drives apoptosis^57,65–67,75,76^. Thus, suppression of ND3 likely represents both a marker and a mediator of BGNps-induced mitochondrial dysfunction in TNBC cells.

Taken together, the coordinated transcriptional reprogramming characterized by p53 upregulation, Bcl-2 downregulation, and ND3 downregulation orchestrates apoptosis in BGNps-treated triple-negative MDA-MB-231 breast cancer cells. While p53 functions as a genomic stress sensor initiating apoptotic signaling, the loss of Bcl-2 removes mitochondrial protection, and suppression of ND3 compromises electron transport, further amplifying ROS generation. This integrated molecular cascade decisively shifts the balance toward intrinsic (mitochondrial) apoptosis, positioning BGNps as a promising therapeutic candidate for TNBC, a subtype that is notoriously resistant to conventional treatments due to defective apoptotic regulation^65–67,75–79^.

As depicted in Fig. 8 this study provides the first experimental evidence that BGNps induce apoptosis in TNBC cells through a multi-step mechanism involving ROS-mediated genomic instability, mitochondrial dysfunction, and transcriptional reprogramming of key apoptotic- and mitochondria-associated regulators. These findings provide a novel mechanistic insights on BGNps-induced anticancer potential and highlight their potential to overcome therapeutic resistance associated with impaired *p53* signaling, a hallmark of TNBC. Despite these promising findings, several limitations should be acknowledged. The study was conducted exclusively in a single in vitro TNBC model, without in vivo confirmation or long-term evaluation of nanoparticle toxicity, biodistribution, or resistance development. Additionally, downstream apoptotic mediators such as caspase activation were not fully characterized, and no direct comparison with standard chemotherapeutic agents was included. Therefore, future research should extend these findings by including normal breast epithelial cells, performing animal studies, and exploring combinatorial or comparative therapeutic approaches to comprehensively assess the safety, selectivity, and translational potential of BGNps in breast cancer treatment.

## Conclusion

Based on the above-discussed findings, it is concluded that BGNps exhibit strong cytotoxic activity against triple-negative MDA-MB-231 breast cancer cells through a multifaceted mechanism involving ROS-induced genomic instability, mitochondrial dysfunction, and transcriptional dysregulation of key apoptotic and mitochondrial genes, ultimately activating intrinsic, *p53*-dependent apoptosis. Hallmark molecular changes included upregulation of apoptotic *p53* and downregulation of mitochondrial *ND3* and anti-apoptotic *Bcl2*, gene expression accompanied by chromatin condensation and nuclear fragmentation, confirming mitochondrial-mediated apoptotic cell death. Collectively, these findings extend the biomedical potential of BGNps beyond their conventional applications in bone regeneration and antimicrobial therapy, highlighting their promise as novel nanotherapeutics for aggressive TNBC and as potential alternatives to conventional chemotherapy.However, the study’s in vitro design represents an important limitation, as no in vivo validation or normal cell selectivity assessment was performed. Moreover, long-term toxicity, resistance development, and detailed mapping of downstream apoptotic mediators e.g., caspase activation remain to be elucidated. Future investigations should therefore include normal breast epithelial cells, animal models, comparative studies with standard chemotherapeutics, and mechanistic evaluations in *p53*-deficient cancers to confirm safety, efficacy, and translational feasibility. Despite these limitations, this work provides a novel mechanistic foundation supporting the further development of BGNps as potential anticancer nanoplatforms for targeted TNBC therapy.

## Data Availability

The datasets used and/or analyzed during the current study are available from the corresponding author on reasonable request.
